# Surveillance for Sickle Cell Disease — Sickle Cell Data Collection Program, Two States, 2004–2018

**DOI:** 10.15585/mmwr.ss7109a1

**Published:** 2022-10-07

**Authors:** Angela B. Snyder, Sangeetha Lakshmanan, Mary M. Hulihan, Susan T. Paulukonis, Mei Zhou, Sophia S. Horiuchi, Karon Abe, Shammara N. Pope, Laura A. Schieve

**Affiliations:** ^1^Andrew Young School of Policy Studies, Georgia Health Policy Center at Georgia State University, Atlanta, Georgia; ^2^Division of Blood Disorders, National Center on Birth Defects and Developmental Disabilities, CDC; ^3^Tracking California Program, Public Health Institute, Richmond, California

## Abstract

**Problem/Condition:**

Sickle cell disease (SCD), an inherited blood disorder affecting an estimated 100,000 persons in the United States, is associated with multiple complications and reduced life expectancy. Complications of SCD can include anemia, debilitating acute and chronic pain, infection, acute chest syndrome, stroke, and progressive organ damage, including decreased cognitive function and renal failure. Early diagnosis, screenings and preventive interventions, and access to specialist health care can decrease illness and death. Population-based public health surveillance is critical to understanding the course and outcomes of SCD as well as the health care use, unmet health care needs, and gaps in essential services of the population affected by SCD.

**Period Covered:**

2004–2018.

**Description of the Program:**

In 2015, CDC established the Sickle Cell Data Collection (SCDC) program to characterize the epidemiology of SCD in two states (California and Georgia). Previously, surveillance for SCD was conducted by two short-term projects: Registry and Surveillance System for Hemoglobinopathies (RuSH), which was conducted during 2010–2012 and included 2004–2008 data, and Public Health Research, Epidemiology, and Surveillance for Hemoglobinopathies (PHRESH), which was conducted during 2012–2014 and included 2004–2008 data. Both California and Georgia participated in RuSH and PHRESH, which guided the development of the SCDC methods and case definitions. SCDC is a population-based tracking system that uses comprehensive data linkages in state health systems. These linkages serve to synthesize and disseminate population-based, longitudinal data for persons identified with SCD from multiple sources using selected *International Classification of Diseases, Ninth Revision, Clinical Modification,* and *Tenth Revision* codes and laboratory results confirmed through state newborn screening (NBS) programs or clinic case reporting. Administrative and clinical data sources include state Medicaid and Children’s Health Insurance Program databases, death certificates, NBS programs, hospital discharge and emergency department records, and clinical records or case reports. Data from multiple sources and years are linked and deduplicated so that states can analyze and report on SCD population prevalence, demographic characteristics, health care access and use, and health outcomes. The SCD case definition is based on an algorithm that classifies cases with laboratory confirmation as confirmed cases and those with a reported clinical diagnosis or three or more diagnostic codes over a 5-year period from an administrative data source as probable cases. In 2019, nine states (Alabama, California, Georgia, Indiana, Michigan, Minnesota, North Carolina, Tennessee, and Virginia) were funded as part of an SCDC capacity-building initiative. The newly funded states developed strategies for SCD case identification and data linkage similar to those used by California and Georgia. As of 2021, the SCDC program had expanded to 11 states with the addition of Colorado and Wisconsin.

**Results:**

During 2004–2018, the cumulative prevalence of confirmed and probable SCD cases identified in California and Georgia was 9,875 and 14,777 cases, respectively. The 2018 annual prevalence count was 6,027 cases for California and 9,141 for Georgia. Examination of prevalence counts by contributing data source during 2014–2018 revealed that each data source captured 16%–71% of cases in California and 17%–87% in Georgia; therefore, no individual source is sufficient to estimate statewide population prevalence. The proportion of pediatric SCD patients (children aged 0–18 years) was 27% in California and 40% in Georgia. The percentage of females with SCD in California and Georgia was 58% and 57%, respectively. Of the cases with SCD genotyping data available (n = 5,856), 63% of patients had sickle cell anemia. SCDC data have been used to directly apprise health care providers and policymakers about health care needs and gaps for patients with SCD. For example, an SCDC Georgia assessment indicated that 10% of babies born during 2004–2016 with SCD lived more than a 1-hour drive from any SCD specialty care option, and another 14% lived within a 1-hour drive of a periodic SCD specialty clinic only. Likewise, an SCDC California assessment indicated that during 2016–2018, most patients with SCD in Los Angeles County lived approximately 15–60 miles from hematologists experienced in SCD care. A surveillance capacity and performance assessment of all 11 SCDC states during 2020–2021 indicated that states differed in the availability of data sources used for SCD surveillance and the time frames for accessing each state data source. Nonetheless, methods for standardizing reporting were developed across all participating states.

**Interpretation:**

This report is the first comprehensive description of CDC’s efforts in collaboration with participating states to establish, maintain, and expand SCD surveillance through the SCDC program to improve health outcomes for persons living with SCD. Findings from California and Georgia analyses highlighted a need for additional SCD specialty clinics. Despite different approaches, expansion of SCDC to multiple states was possible using standardized, rigorous methods developed across all participating states for reporting on disease prevalence, health care needs and use, and deaths.

**Public Health Action:**

Findings from surveillance can be used to improve and monitor care and outcomes for persons with SCD. These and other SCDC analyses have had a role in opening new SCD clinics, educating health care providers, developing state health care policies, and guiding new research initiatives. Public health officials can use this report as a guiding framework to plan or implement surveillance programs for persons with SCD. Both data-related activities (data sources; patient identifiers; and obtaining, transferring, and linking data) and the administrative considerations (stakeholder engagement, costs and resources, and long-term sustainability) are crucial to the success of these programs.

## Introduction

### Overview of Sickle Cell Disease

Sickle cell disease (SCD), an inherited blood disorder that causes abnormal hemoglobin production in affected persons, is associated with multiple complications and reduced life expectancy. SCD affects approximately 100,000 persons in the United States (*1*). Complications of SCD vary for each person, and the life span of those with the most severe form is on average 20–30 years shorter than for the general U.S. population (*2*,*3*). SCD affects millions of persons throughout the world and is particularly common among those whose ancestors are from sub-Saharan Africa, Spanish-speaking regions in the Western Hemisphere, Saudi Arabia, India, and Mediterranean countries (*4*). The relatively high prevalence of SCD in the United States is a result of slave trade as well as migration and settlement by persons from countries where the disease is more common but potentially undiagnosed (*5*).

SCD comprises a group of disorders characterized by the presence of at least one allele (an alternate form or versions of a gene) that causes sickling, hemoglobin S (HbS; p.Glu6Val in the *HBB* gene), and a second *HBB* gene pathogenic variant resulting in abnormal hemoglobin polymerization (*6*). The *HBB* gene provides instructions for making a protein called beta-globin (β-globin). HbS changes flexible red blood cells into rigid, sickle-shaped cells (i.e., sickling) that can obstruct blood flow resulting in pain and organ damage (*7*). The most common form of SCD occurs in persons homozygous for the HbS variant (HbSS). Other forms of SCD include coinheritance of the HbS variant with another β-globin gene variant, including hemoglobin C (HbSC), hemoglobin D-Los Angeles/Punjab, hemoglobin O-Arab, or β-thalassemia (HbS/β^0^-thalassemia and HbS/β^+^-thalassemia). The HbSS and HbS/β^0^-thalassemia genotypes are usually the most severe forms of SCD and are collectively referred to as sickle cell anemia (*8*,*9*). SCD is inherited in a classic autosomal recessive pattern. Inheritance of one abnormal and one normal allele confers sickle cell trait, a carrier state in which persons rarely exhibit clinical symptoms such as pain crises (*10*), whereas inheritance of two abnormal alleles, one from each parent, results in SCD. The disorder is characterized by varying amounts of chronic hemolytic anemia, debilitating acute and chronic pain, infection, acute chest syndrome, stroke, progressive organ damage, and decreased cognitive function (*11*,*12*).

### Clinical Care and Course of SCD

Research suggests that patients with SCD benefit from care provided by a physician who is a specialist in the treatment of SCD (*13*–*15*). Management of SCD is focused on preventing and treating pain episodes and complications. Prevention strategies include lifestyle behaviors such as maintaining adequate fluid intake, regular physical activity, and avoiding extreme temperatures; medical screenings such as transcranial Doppler (TCD) ultrasound screenings in children; and medical interventions such as vaccines and penicillin prophylaxis to prevent infections and regular blood transfusions to prevent stroke in those with abnormal TCD screening results (*16*). In addition, medications might prevent or reduce the occurrence of red blood cell sickling in patients with severe disease; however, medication side effects (e.g., headache, nausea, and nail or skin hyperpigmentation), treatment intensity, and other patient and provider barriers have reduced the use of these medications (*17*–*20*). Management of pain crises can include administration of opioids, intravenous fluids, and pain-reducing medications; severe pain crises often require hospitalization. Curative treatments for SCD include stem cell transplant and emerging gene therapies. Although these treatments offer great promise, stem cell transplants can be associated with severe, life-threatening complications, and the safety of gene therapies is under investigation (*21*–*23*).

The risks for adverse health outcomes in persons with SCD are compounded by racial, socioeconomic, and health care disparities. Approximately 90% of persons with SCD in the United States are Black or African American (Black). Hispanic or Latino (Hispanic) persons also make up a notable proportion. Persons with SCD often face barriers to care because of both structural racism (e.g., policies that have led to unequal opportunities in housing and employment, inconsistent health insurance, and lack of funding for the development of treatments for SCD) and interpersonal racism (e.g., racist overtones and bias toward patients with SCD seeking care for pain crises, which often results in inadequate care and continued suffering) (*2*,*24*).

SCD complications can adversely affect educational achievement and employment and, subsequently, a person’s ability to seek and receive care (*25*,*26*). Challenges to receiving appropriate health care for SCD are compounded by the lack of providers with expertise in treating SCD and difficulties in care coordination between primary and subspecialty services (*27*,*28*). Consequently, persons with SCD might delay seeking care, and emergency department visits are frequent among these patients. These factors result in costly dependence on acute care from hospitals and emergency departments, disrupting continuity of care and leading to higher rates of readmission (*29*,*30*).

### History of SCD Surveillance in the United States

In 2015, CDC established the Sickle Cell Data Collection (SCDC) program to characterize the epidemiology of SCD in two states (California and Georgia). Before SCDC was established, surveillance for SCD was conducted by two short-term state surveillance projects: Registry and Surveillance System for Hemoglobinopathies (RuSH) and Public Health Research, Epidemiology, and Surveillance for Hemoglobinopathies (PHRESH) (Table 1). The RuSH and PHRESH projects, which provided important insights about the populations living with SCD and the complexities of tracking SCD statewide, guided the development of SCDC.

**TABLE 1:** History of sickle cell disease surveillance in the United States

Characteristic	RuSH (2010–2012)	PHRESH (2012–2014)	SCDC (2015–present)
**Purpose**	Pilot program to develop a system to identify and collect data on persons living with SCD in the participating states	**California and Georgia:** To evaluate and validate the data collected via RuSH **Mississippi:** To identify and collect data on persons living with SCD	To continue the efforts of RuSH and PHRESH; build capacity in other states for SCD surveillance; and study trends in diagnosis, treatment, and health care access to improve the lives of persons with SCD
**Years of data**	2004–2008		2004–2018
**Data sources**	Newborn screening Vital records (birth and death records) Hospital discharge Emergency department Clinical records State Medicaid claims		Newborn screening Vital records (death records) Hospital discharge Emergency department Clinical records State Medicaid claims
**Participating states**	California, Florida, Georgia, Michigan, New York, North Carolina, and Pennsylvania	California, Georgia, and Mississippi	California (since 2015); Georgia (since 2016); and Alabama, Colorado, Indiana, Michigan, Minnesota, North Carolina, Tennessee, Virginia, and Wisconsin (since 2020–2021)
**Funding source**	National Institutes of Health National Heart, Lung, and Blood Institute	**CDC:** Office of the Director Division of Blood Disorders (discretionary)	Funding has varied throughout the project. **CDC:** Office of the Director National Center on Birth Defects and Developmental Disabilities Deputy Director for Non-Infectious Diseases Division of Blood Disorders (discretionary and congressional) **CDC Foundation:** Bioverativ Doris Duke Charitable Foundation Global Blood Therapeutics Novartis Foundation Pfizer **U.S. Department of Health and Human Services:** Office of Minority Health Centers for Medicare & Medicaid Services Food and Drug Administration
**Funding amount**	Project total: $2.2 million	Project total: $1.4 million	**2020:** 11 states at $2.4 million, approximately $220,000 per state (range: $110,000–$370,000) **2021:** 11 states at $3.4 million, approximately $310,000 per state (range: $250,000–$380,000) **2022:** 11 states at $3.6 million, approximately $325,000 per state (range: $250,000–$416,000)
**Congressional funds**	$0	$0	**FY 2020 and before:** $0 **FY 2021:** $2 million **FY 2022:** $3 million

**Abbreviations:** FY = fiscal year; PHRESH = Public Health Research, Epidemiology, and Surveillance for Hemoglobinopathies; RuSH = Registry and Surveillance System for Hemoglobinopathies; SCD = sickle cell disease; SCDC = Sickle Cell Data Collection.

#### RuSH

RuSH (2010–2012) was established to determine the state-specific, population-based numbers of persons with SCD in selected states and increase knowledge and awareness about health care use and outcomes. Through a work group of clinicians and public health professionals, investigators developed a case definition (Tables 2 and 3) to help standardize the collection of SCD data across the states. RuSH also laid the groundwork for states, with the help of data sharing agreements and letters of support, to capture patient data through partnerships with various public health government agencies, specialty treatment centers, and community entities.

**TABLE 2:** Original Registry and Surveillance System for Hemoglobinopathies case definition and revised Sickle Cell Data Collection case definition

Case classification	RuSH case definition (2010–2012)	SCDC case definition (2015–present)
**Level 1: Confirmed case**	CLIA-certified laboratory result of SCD* reported by a state NBS program with confirmatory testing ***or*** Clinical diagnosis by a physician with documented confirmatory CLIA-certified laboratory testing after the newborn period	CLIA-certified laboratory result of SCD* reported by a state NBS program with confirmatory testing ***or*** Clinical diagnosis by a physician with documented confirmatory CLIA-certified laboratory testing after the newborn period
**Level 2: Probable case**	CLIA-certified laboratory result of SCD reported by a state NBS program without report of confirmatory testing ***or*** SCD ICD-CM code at two or more separate health care encounters ***plus*** One or more SCD-associated complication,^†^ treatment,^§^ or procedure^¶^	CLIA-certified laboratory result of SCD reported by a state NBS program without report of confirmatory testing ***or*** Clinical diagnosis by a physician without documented confirmatory CLIA-certified laboratory testing after the newborn period ***or*** SCD ICD-CM code (excluding sickle cell trait) on three or more separate health care encounters during a 5-year period
**Level 3: Possible case**	Sickle cell trait ICD-CM code at two or more separate health care encounters ***plus*** One or more SCD-associated complication, treatment, or procedure ***or*** SCD ICD-CM code for a single health care encounter	SCD ICD-CM code (including sickle cell trait) for one or two health care encounters

**Abbreviations:** CLIA = Clinical Laboratory Improvement Amendments; ICD-CM = *International Classification of Diseases, Clinical Modification;* NBS = newborn screening; RuSH = Registry and Surveillance System for Hemoglobinopathies; SCD = sickle cell disease; SCDC = Sickle Cell Data Collection.

* Includes hemoglobin SS, hemoglobin S/β^0^-thalassemia, hemoglobin SC, hemoglobin S/β^+^-thalassemia, and other compound heterozygous forms of SCD.

^†^ Chronic renal failure, proteinuria, pneumonia, acute chest syndrome, pulmonary hypertension, stroke (ischemic or hemorrhagic), transient ischemic attack, seizures, intracranial bleeding, priapism, iron overload, gallstones, cholelithiasis, cholecystitis, avascular necrosis, retinal disease, splenomegaly, splenic sequestration, hypersplenism, leg ulcers, dactylitis, and osteomyelitis.

^§^ Hydroxyurea, parenteral analgesics, iron chelators, erythropoietin, and folic acid.

^¶^ Red cell transfusion, red cell exchange, splenectomy, cholecystectomy, and transcranial Doppler.

**TABLE 3:** *International Classification of Diseases, Ninth Revision, Clinical Modification*, *and International Classification of Diseases, Tenth Revision, Clinical Modification* codes used to identify persons with sickle cell disease within administrative data sources

ICD-9-CM		ICD-10-CM	
282.41	Sickle cell thalassemia without crisis	D57	Sickle cell disorders
282.42	Sickle cell thalassemia with crisis	D57.0X	Sickle cell anemia with crisis
282.6	Sickle cell disease, unspecified	D57.1X	Sickle cell anemia without crisis
282.61	Sickle cell/hemoglobin-SS disease without crisis	D57.2X	Double heterozygous sickling disorders (hemoglobin S/C, hemoglobin S/D, hemoglobin S/E, sickle cell thalassemia)
282.62	Sickle cell/hemoglobin-SS disease with crisis	D57.4X	Sickle cell thalassemia
282.63	Sickle cell/hemoglobin-C disease without crisis	D57.8X	Other sickle cell disorders
282.64	Sickle cell/hemoglobin-C disease with crisis	D57.3	Sickle cell trait
282.68	Other sickle cell disease without crisis		
282.69	Other sickle cell disease with crisis		
282.5	Sickle cell trait		

**Abbreviations:** ICD-9-CM *= International Classification of Diseases, Ninth Revision, Clinical Modification*; ICD-10-CM = *International Classification of Diseases, Tenth Revision, Clinical Modification*.

RuSH used data from persons with SCD who received care and services during 2004–2008. Newborn screening (NBS) records, hospital discharge data (including emergency department records), death records, clinical records, and state Medicaid claims were used for both case identification and as sources of demographic, medical, and health care use data. Each state used a unique combination of data sources for the project, depending on the data sets they could access. Full methods and results from six of the seven participating RuSH states have been reported previously (*31*). The development of RuSH built many new partnerships and coalitions. State health department employees, health care providers, academic institutions, community organizations, patients, and families were all important contributors to the program. Products, peer-reviewed publications, and accomplishments from RuSH are available at https://www.cdc.gov/ncbddd/hemoglobinopathies/surveillance-history.html.

#### PHRESH

PHRESH (2012–2014) was designed to further validate SCD surveillance case definitions and data integration methods and disseminate SCD surveillance data. PHRESH refined the SCD surveillance definition and demonstrated the use and challenges of studying SCD using administrative data. Similar to RuSH, PHRESH used retrospective data from persons with SCD who received care and services during 2004–2008.

#### SCDC

SCDC is a population-based longitudinal surveillance program with methods built on the foundations developed during the RuSH and PHRESH limited-term surveillance programs. SCDC expanded surveillance efforts by including additional years of data and allowing for new population-based analyses of various health topics, such as geographic distribution of SCD cases and specialty clinics, SCD in Hispanic persons, older patients with SCD, use of health care services, and the transition from pediatric to adult care.

Funding sources and support for SCD surveillance has varied over the years, including funding through the CDC Foundation; limited term federal funding allocations through CDC, the U.S. Department of Health and Human Services, and the Centers for Medicare & Medicaid Services; and most recently, designated congressional appropriation. Beginning in 2015, two programs were funded to further develop and implement SCDC in their respective states: California (Public Health Institute’s Tracking California program in partnership with the California Department of Public Health) and Georgia (Georgia State University’s Georgia Health Policy Center). These two state-based SCDC programs are the most mature and robust; therefore, surveillance methods, data highlights, and examples of how the SCD surveillance data have been used to inform policy or practice from these states are described in this report.

### SCD and Surveillance

Ongoing public health surveillance is critical to understanding the clinical course and outcomes of SCD and is vital for public health planning and patient, family, and provider education. Although SCD is a genetic disease diagnosed by NBS in the United States, such screening was not widespread until the 1990s and does not account for persons born outside of the United States (*32*). Disease registries follow persons with SCD over time to improve their care and clinical outcomes; however, registries are usually limited to a specific health care provider or group of providers. Therefore, disease registries miss persons who have never been seen at that facility, either because they have received care elsewhere or have had limited access to care because of distance and transportation challenges, lack of insurance coverage, or both (*33*). No national population-based surveillance system exists for SCD (*34*). This report describes CDC’s SCDC program and the surveillance projects that preceded it, highlights recent data (2004–2018) from SCDC, and describes SCDC capacity-building activities to expand the number of participating states. Public health officials can use this report as a guiding framework to plan or implement SCD surveillance programs.

## Methods

### Data Sources

Through RuSH, PHRESH, and SCDC, the California and Georgia programs have received surveillance data from various sources through longstanding collaborations with data stewards from their state agencies and clinic sites (Table 4). Although most data sources for SCDC were the same as those included in their earlier surveillance programs, both California and Georgia added new clinic reporting sites since RuSH. Further information on the participating clinic sites in California and Georgia is available (Supplementary Material 1, https://stacks.cdc.gov/view/cdc/120911).

**TABLE 4:** Sickle Cell Data Collection data sources, data elements, and data use for sickle cell disease surveillance

Data source	Agency	Data element	Data use for SCD surveillance
Newborn screening	Public health department	Personal identifiers, demographics, geographic information, screening, and laboratory-confirmed genotype results	NBS data provide SCD incidence each year. NBS information linked to other data sources can enable confirmation that the person has SCD and the disease variant.
Death certificate	Public health department	Date of death, place of death, underlying and contributing causes of death, personal identifiers, and demographics	To link to state SCD cases to identify deaths in this cohort.
Hospital discharge data and emergency department data	Different for each state (e.g., Georgia data obtained from the Georgia Hospital Association and California data obtained from the Office of Statewide Health Planning and Development)	Date of service, diagnoses, procedures, site of service, personal identifiers, demographics, and payer types	Encounter data provide both evidence of case status and information about health care use, diagnosis codes, procedure codes, and geography of the person at time of encounter.
Clinical records or case reports	Clinic sites or comprehensive sickle cell treatment centers	Laboratory-confirmed genotype, personal identifiers, and most recent date of service	To confirm SCD status and genotype and to identify who is being cared for by SCD specialists
State Medicaid and CHIP claims and eligibility (enrollment) data	Different for each state (e.g., Georgia data obtained from the Department of Community Health and California data obtained from the Department of Health Care Services)	Enrollment information, including personal identifiers and demographic, geographic, and eligibility information; diagnosis and procedure codes from claims data, including inpatient, outpatient, and pharmacy claims; and provider information	Claims data provide both evidence of case status and information about health care use, prescriptions, diagnosis codes, procedure codes, and geography of the person at time of encounter. Medicaid and CHIP eligibility data provide demographic and geographic information. In certain states, these data might also allow evaluation of cost information and information about the providers overseeing the medical care of the population affected by SCD.
Other sources	National Provider Index		To detail provider information (e.g., provider specialty and location)
	American Community Survey; Area Resource File, U.S. Census Bureau data		County and census geography information
	State immunization information system		Date and type of vaccinations received by provider type and location

**Abbreviations:** CHIP = Children’s Health Insurance Program; NBS = newborn screening; SCD = sickle cell disease.

Even for previously used data sources, the process for reestablishing data sharing agreements with each data steward required a substantial time investment. Data sharing agreements were rewritten to justify each data element and cover ongoing surveillance efforts over multiple years rather than a one-time pilot project. The data elements received from the various data sources include identifiers allowing the data sources to be linked and cases deduplicated.

SCDC programs in California and Georgia identify cases through NBS, hospital discharge and emergency department records, clinic provider records, and state Medicaid and Children’s Health Insurance Program (CHIP) claims data. Both California and Georgia also receive supplementary data from vital records, and Georgia receives data from the state immunization information system. For all identified cases, states continue to receive information in subsequent years from hospital discharge, emergency department, and outpatient (Medicaid only) records. Surveillance data also have been linked to publicly available data sources (e.g., American Community Survey and National Provider Index) using provider or geographic identifiers to better understand the types of providers being accessed and the social determinants of health in affected communities. In addition to the data for cases identified under the SCDC program, both California and Georgia have retained access to case data collected through the RuSH and PHRESH projects.

### Surveillance Case Definition

The SCDC surveillance case definition was developed based on a validation study of the RuSH case definition (*35*). However, there are multiple differences (Tables 2 and 3).

#### Confirmed Case Definition

The confirmed SCD case definition used in SCDC remains the same as it was in RuSH. Laboratory confirmation is required for a case to be classified as confirmed.

#### Probable Case Definition

The probable case definition was updated to expand and simplify the algorithm used to identify SCD cases from *International Classification of Diseases, Ninth Revision, Clinical Modification,* and *Tenth Revision* (ICD-CM) codes in administrative data sources and add clinical case reports without laboratory confirmation to the SCD probable case definition. A validation study indicated that the new algorithm to identify probable cases substantially improved the sensitivity (96.0% versus 85.8%) and negative predictive value (68.2% versus 38.2%) for identifying cases of SCD while maintaining a positive predictive value (97.4% versus 97.4%) and a specificity (76.5% versus 79.0%) that were similar to the original algorithm. Both confirmed cases and probable cases are included in SCDC reports (*35*).

#### Possible Case Definition

The possible case definition also was simplified and updated. Possible cases in SCDC are defined as those with either an SCD ICD-CM code or sickle cell trait ICD-CM code on a single health care encounter. The possible case definition is the most inclusive algorithm producing the largest number of potential cases when applied to administrative data. Possible cases are not reported in SCDC surveillance reports because the validation study found that only 28 of the 128 patients meeting the possible case definition had SCD (*35*).

### Data Linkage Strategies and Deduplication

**FIGURE 1:**
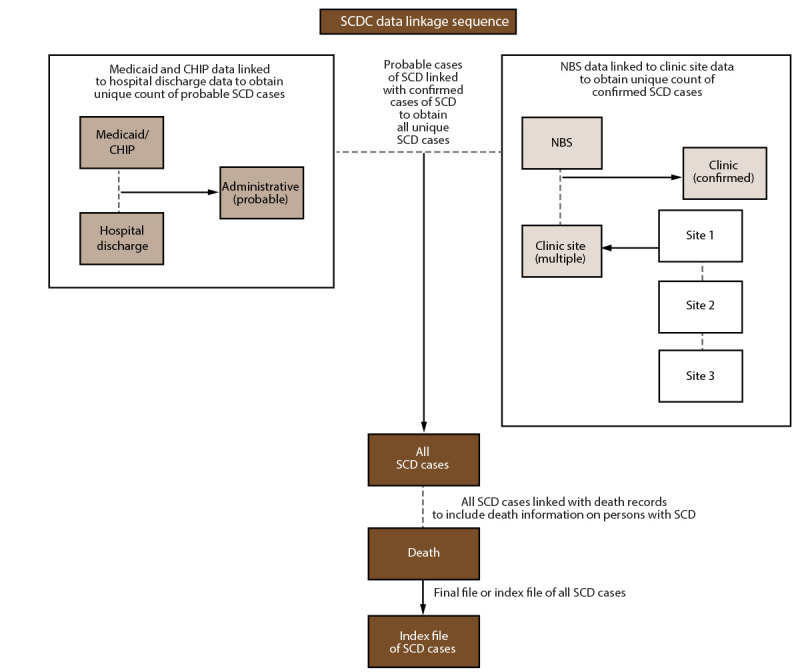
Sickle Cell Data Collection surveillance data linkage and deduplication process **Abbreviations:** CHIP = Children’s Health Insurance Program; NBS = newborn screening; SCD = sickle cell disease; SCDC = Sickle Cell Data Collection.

After data are received from all data sources, an iterative process of deduplication and linkage is followed to create a master index file for each case in the data system (Figure 1). States use different methods for linking (both probabilistic and deterministic), depending on the identifiers available in each data set (e.g., name, Social Security number, and date of birth). Both California and Georgia programs use SAS software (version 9.4; SAS Institute) for analysis; in addition, California uses a custom Java application (version 8; Oracle) to conduct the data linkage. Most linkage processes require a level of manual review. Although the linkage process differs in each state, the process typically involves a loosely ordered set of strategies, as follows:

1. Individual data sets are cleaned and deduplicated as a first step to perform quality checks on the full data set. This process includes a thorough review of the data, fixing records with missing information and thereby ensuring that the same patient is not recorded multiple times in the data set.
2. Data sets containing confirmed cases (clinical and NBS data sets) are linked to create an index file containing a unique identifier for each case. This process continues until those data sets containing confirmed cases are linked, deduplicated, and indexed.
3. A similar process is followed for administrative data sets in which persons with one or more SCD ICD-CM codes in hospital, emergency department, or Medicaid data sets are linked across data sets, deduplicated, and indexed.
4. The confirmed cases are then linked and merged to the persons identified in the administrative data sets. This step results in a matched data set with confirmed cases linked to administrative data, when possible, and a data set of unmatched persons from administrative data sets only.
5. The SCDC case definition algorithm is then applied to this data set of unmatched persons to classify each as a probable or possible SCD case. Although not used for SCDC analyses, the possible cases are retained because they might link to data collected in the future and be reclassified as probable or confirmed cases.
6. The final index file contains deduplicated cases by case status (confirmed or probable) along with the associated personally identifiable information and a unique identifier that links back to the original data sets, allowing access to additional variables for analysis.
7. Supplementary data (data that are not used for case finding but provide additional information on identified cases) are typically linked after all cases are indexed. An example of supplementary data is vital death records that help identify deaths within the data set.

### Data Collection

**FIGURE 2:**
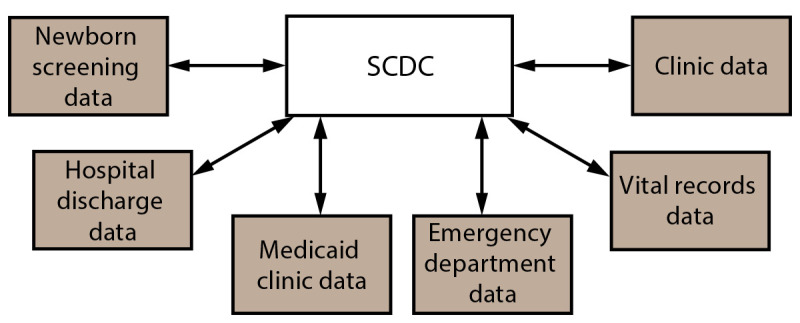
Sickle Cell Data Collection as a centralized index file connected to source data sets* **Abbreviation:** SCDC = Sickle Cell Data Collection. * Each data set depicted retains its original file structure and data elements.

The final SCDC data structure is a hub and spoke model with the centralized index file (the hub) connected to the source data sets (the spokes), with each retaining its original file structure and data elements (Figure 2). This flexible structure allows for claims data collected longitudinally to be combined with point-in-time data sources such as NBS. Therefore, each state’s index file includes all deduplicated cases over a specified time. Using the index file, demographics for a case, such as a patient’s race and ethnicity, can be pulled from the specific data source each state deems most reliable (e.g., a state might consider demographics drawn from NBS data to be more reliable than emergency department encounter data). Depending on the question to be answered using the surveillance data, a subset of the data is extracted that contains the specific data elements (pulled from the index file and source data sets) necessary to answer the question. In California and Georgia, more than 15 years of retrospective data (2004–2018) have been collected and linked to ensure comprehensive case ascertainment, allow for longitudinal follow-up, and provide data for trend analyses. As additional years of data have been added, California and Georgia have developed methods to account for migration in and out of each state when estimating annual prevalence from case counts (https://www.cdc.gov/ncbddd/hemoglobinopathies/scdc-data.html).

### SCDC Expansion

In 2019, seven states (Alabama, Indiana, Michigan, Minnesota, North Carolina, Tennessee, and Virginia) received funding as part of an SCDC capacity-building initiative for new states to develop strategies for SCD case identification and data linkage similar to those used by the established programs in California and Georgia. The California and Georgia programs also were funded in this capacity-building project to collaborate with CDC to provide technical assistance to the new states through the development and implementation of virtual and in-person training sessions and other mechanisms for knowledge sharing and relationship building. As funding became available in 2020 and 2021, CDC expanded the SCDC network to include Colorado and Wisconsin, for a total of 11 states participating in SCDC.

### Analysis

Prevalence estimates and population characteristics, recent analyses that guided health policy, and summary assessments of SCDC capacity-building and performance-reporting activities are described in this report. This activity was reviewed by CDC and was conducted consistent with applicable federal law and CDC policy.*

#### Prevalence Estimates and Population Characteristics

Cumulative SCD population prevalence counts using data during 2004–2018 are presented by case status (confirmed or probable) and data source alongside annual population prevalence counts using 2018 data, the most recent surveillance year for the California and Georgia programs. SCD subtype and demographic distributions also are presented by data source and state.

#### Analyses that Informed Health Care Providers and Policymakers

Projects are described that illustrate how SCDC data are used to directly apprise health care providers and policymakers about SCD health care needs and gaps. To study access to specialized pediatric SCD care, Georgia used street address information from NBS records during 2004–2016, paired with address information from the pediatric SCD treatment and comprehensive treatment centers with daily access and the sickle cell specialty clinics with periodic access, to determine access for newborns to SCD care within a 1-hour drive. Similarly, California used both patient and hematologist address information from Los Angeles County available in Medi-Cal to compute distance to hematologists with experience caring for patients with SCD. SCDC teams used ArcGIS software (desktop version 10.7.1; Esri) with street-map premium data for the analyses.

Both California and Georgia also developed a data brief that assessed percentages of hydroxyurea prescriptions filled during 2006–2018 (using National Drug Codes available in pharmacy claims) among their Medicaid and CHIP recipients who had an established indication for hydroxyurea use (i.e., at least one occurrence of acute chest syndrome or three acute pain episodes determined using ICD-CM codes). This project was developed to monitor adherence to best practices in SCD treatment over time.

#### SCD Surveillance Process Assessment

Descriptive findings from a process assessment first implemented in 2020 and SCDC performance reporting from all participating SCDC states are presented. Information on data source availability is described for all 11 SCDC states. Data sources are classified as core, defined as critical to primary surveillance objectives and case findings (e.g., NBS, hospital and emergency department encounter data, and all payer health care claims) or supplemental, defined as ancillary to primary objectives and adding additional information but no new cases (e.g., vital records death files provide information on the circumstances of death, birth certificates provide additional information on newly identified cases and birth location, and National Provider Index data offer additional information on provider specialty).

## Results

### Prevalence Estimates and Population Characteristics

During 2004–2018, the cumulative prevalence of confirmed and probable SCD cases identified in California and Georgia was 9,875 and 14,777 cases, respectively. The 2018 annual prevalence count was 6,027 for California and 9,141 for Georgia (Table 5). Examination of prevalence counts by contributing data source revealed that during 2014–2018, each data source captured 16%–71% of cases in California and 17%–87% in Georgia; thus, none of the sources is sufficient individually to estimate population prevalence. Medicaid data are an important source of longitudinal data, contributing 38,841 person-years in California and 78,382 person-years in Georgia to the surveillance data. Similarly, hospital discharge and emergency department data provide 32,926 person-years of data in California and 83,114 person-years of data in Georgia to study acute health care use over time.

**TABLE 5:** Number of confirmed and probable cases of sickle cell disease, by data source — Sickle Cell Data Collection, California and Georgia, 2004–2018

Data source	2004–2018					2018 annual prevalence*			
	Confirmed SCD (no.)	Probable SCD (no.)	Total (no.)	% of deduplicated total	Person-years of data	Confirmed SCD (no.)	Probable SCD (no.)	Total (no.)	% of deduplicated total
**California**									
Newborn screening	1,612	12	**1,624**	16.4	—^†^	1,014	2	**1,016**	16.9
Clinic sites^§^	2,592	147	**2,739**	27.8	—	1,734	59	**1,793**	29.8
Medicaid and CHIP	2,058	4,441	**6,499**	65.8	38,841	1,795	3,325	**5,120**	84.9
Hospital discharge	1,812	5,206	**7,018**	71.1	32,926	1,502	3,189	**4,691**	77.8
**Deduplicated total**	**3,389**	**6,486**	**9,875**	**100.0**	**—**	**2,119**	**3,908**	**6,027**	**100.0**
**Georgia**									
Newborn screening	2,359	128	**2,487**	16.8	—	1,882	43	**1,925**	21.1
Clinic sites^¶^	8,150	129	**8,279**	56.0	—	5,743	67	**5,810**	63.6
Medicaid and CHIP	6,184	3,319	**9,503**	64.3	78,382	4,653	2,009	**6,662**	73.0
State health benefit plan**	249	191	**440**	3.0	—	—	—	**—**	—
Hospital discharge	7,324	5,473	**12,797**	86.6	83,114	5,427	2,918	**8,345**	91.3
**Deduplicated total**	**8,403**	**6,374**	**14,777**	**100.0**	**—**	**5,856**	**3,285**	**9,141**	**100.0**

**Abbreviations:** CHIP = Children’s Health Insurance Program; SCD = sickle cell disease.

* 2018 annual prevalence includes SCDC confirmed and probable cases that had evidence of residency within the respective state in 2018 (defined as one or more health care encounters within the state in 2018 or later).

^†^ Person-years is only reported for the individual data sources contributing data on the same persons over time because those sources contribute both person counts and time elements to surveillance.

^§^ Clinic data sites in California: University of California San Francisco Zuckerberg General Hospital; University of California San Francisco Benioff Children’s Hospital Oakland; Lucille Packard Children’s Hospital Stanford; University of California Davis Medical Center; Children’s Hospital Orange County, Center for Inherited Blood Disorders; Children’s Hospital Los Angeles; University of California San Diego Rady Children’s Hospital; and Valley Children’s Hospital.

^¶^ Clinic data sites in Georgia: Georgia Comprehensive Sickle Cell Center at Grady Memorial Hospital; Sickle Cell Disease Program at Children’s Healthcare of Atlanta; Augusta University Sickle Cell Center; and Memorial Health Dwaine and Cynthia Willett Children’s Hospital of Savannah.

** Georgia state health benefit plan data were collected for 2004–2008 only.

The 2018 annual SCD confirmed case prevalence reflects that 63% of persons had sickle cell anemia (i.e., HbSS or HbS/β^0^-thalassemia) (California and Georgia: 63%); 8% had HbS/β^+^-thalassemia (California and Georgia: 8%); 27% had HbSC (California: 26%; Georgia: 28%); and 2% had another form of SCD (California: 3%; Georgia: 1%). The pediatric SCD population (aged 0–18 years) in California was 27% of the total SCD population and 40% in Georgia. In California, 58% of persons with SCD were female, and in Georgia, 57% of persons with SCD were female (Table 6).

**TABLE 6:** Number of persons with sickle cell disease, by data source, genotype, sex, and age group — California and Georgia, 2018

Data source	Confirmed SCD					Probable SCD	Total			
	No.	SS/Sβ^0^-thal (%)	Sβ^+^-thal (%)	SC (%)	Other SCD (%)	Unknown genotype (no.)	No.	Male (%)	Female (%)	Aged 0–18 yrs* (%)
**California**										
Newborn screening	1,014	60.0	9.7	26.2	4.1	2	**1,016**	**53.0**	**47.0**	**94.9**
All clinical sources	1,734	65.1	7.0	24.9	2.9	59	**1,793**	**49.3**	**50.7**	**43.5**
Medicaid or CHIP	1,795	63.2	7.5	26.1	3.2	3,325	**5,120**	**41.9**	**58.1**	**28.0**
Hospital discharge	1,502	68.4	6.5	23.3	1.9	3,189	**4,691**	**40.4**	**59.6**	**17.6**
**Deduplicated total**	**2,119**	**63.3**	**7.5**	**26.0**	**3.3**	**3,908**	**6,027**	**42.2**	**57.8**	**27.1**
**Georgia**										
Newborn screening	1,882	59.9	8.3	30.0	1.8	43	**1,925**	**50.4**	**49.6**	**100.0**
All clinical sources	5,743	63.7	7.6	27.5	1.2	67	**5,810**	**48.9**	**51.1**	**53.1**
Medicaid or CHIP	4,653	64.2	7.5	27.0	1.3	2,009	**6,662**	**42.7**	**57.3**	**45.1**
Hospital discharge	5,427	64.4	7.6	26.9	1.1	2,918	**8,345**	**43.1**	**56.9**	**37.5**
**Deduplicated total**	**5,856**	**63.4**	**7.7**	**27.7**	**1.2**	**3,285**	**9,141**	**43.2**	**56.8**	**39.6**

**Abbreviations:** CHIP = Children’s Health Insurance Program; SCD = sickle cell disease; thal = thalassemia.

* Age as of December 31, 2018.

### Analyses that Informed Health Care Providers and Policy

**FIGURE 3:**
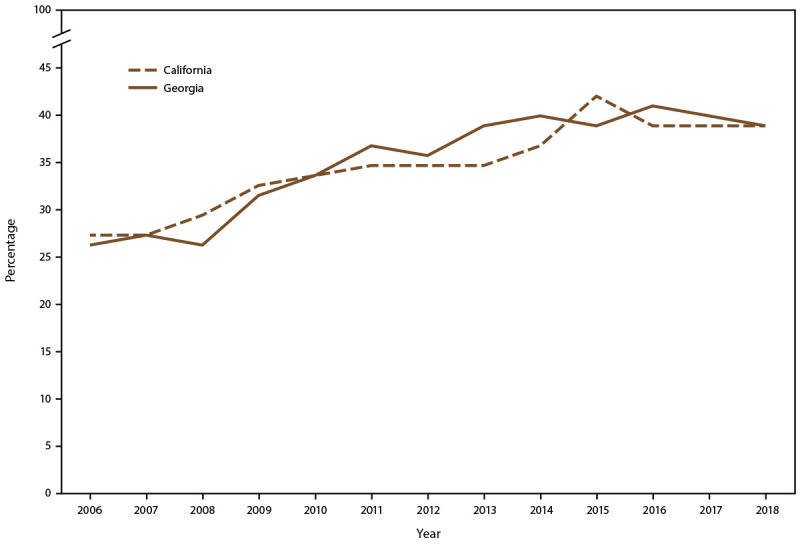
Percentage of Medicaid beneficiaries with sickle cell disease who filled one or more prescriptions for hydroxyurea, by year — Sickle Cell Data Collection, California and Georgia, 2006–2018

Of the 2,006 newborns with SCD born in Georgia during 2004–2016 with available address information, 10% lived more than a 1-hour drive from any specialty care option, and another 14% lived within a 1-hour drive of a specialty clinic with periodic access only (*36*). California SCDC data during 2016–2018 indicated that most patients covered by Medicaid in Los Angeles County (n = 1,800) were located approximately 15–60 miles from hematologists experienced in SCD care. Additional information on the geographic distribution of SCD clinics in California and Georgia is available (Supplementary Figures 1 and 2, https://stacks.cdc.gov/view/cdc/120911). During 2006–2018, percentages of hydroxyurea prescriptions filled for Medicaid recipients with SCD meeting clinical indications (history of acute chest syndrome or three or more acute pain episodes) increased from 26% to 37% in California and from 25% to 37% in Georgia (Figure 3).

### SCD Surveillance Process Assessment

During surveillance capacity and performance assessments conducted during 2020–2021, all participating states provided information on their existing or baseline infrastructure that could support SCD statewide surveillance. Most states had existing relations with their public health departments and Medicaid agencies. The lead agency in many states was the public health department, with experience performing surveillance on other conditions and linking data from Medicaid, clinical partners, payor databases, or emergency department or hospital discharge data. The involvement of the public health department meant that multiple states could receive exemptions from their state institutional review boards for performing SCD surveillance activities (Table 7). Multiple states also had relations with the Medicaid agencies, including researchers who have used that data, had active data sharing agreements, or both.

**TABLE 7:** States granted public health surveillance exemption and institutional review board approval for Sickle Cell Data Collection statewide surveillance

SCDC state	Public health surveillance exemption	Administrator of IRB approval or IRB exemption
Alabama	Exempt	University or academic institution
California	Not exempt. SCDC in California is a collaboration between PHI and CDPH, and certain nonsurveillance activities required review by both PHI and CDPH IRBs.	Other state IRB (California Committee for the Protection of Human Subjects)
Colorado	Exempt	University or academic institution
Georgia	Exempt	University or academic institution
Indiana	Exempt	No IRB needed because of public health exemption status
Michigan	Exempt	State public health department and university or academic institution
Minnesota	Exempt	No IRB needed because of public health exemption status
North Carolina	Not exempt. SCDC in North Carolina is a joint co-led collaboration between Duke University and NCDHHS. Duke University is subcontracted to perform SCDC activities.	University or academic institution
Tennessee	Not exempt	State public health department
Virginia	Exempt	State public health department
Wisconsin	Exempt	University or academic institution

**Abbreviations:** CDPH = California Department of Public Health; IRB = institutional review board; NCDHHS = North Carolina Department of Health and Human Services; PHI = Public Health Institute; SCDC = Sickle Cell Data Collection.

The process assessment indicated that states differed in the ways they accessed core and supplemental data sources for SCD surveillance purposes (Table 8). This can be attributed to data source and data element availability and the specific time frame each state data source is available.

**TABLE 8:** Data sources used by the Sickle Cell Data Collection state programs for sickle cell disease surveillance, by state

Data source	Alabama	California	Colorado	Georgia	Indiana	Michigan	Minnesota	North Carolina	Tennessee	Virginia	Wisconsin
Newborn screening and genetic counseling data	C	C	C	C	C	C	C	C	C	C	C
Vital records birth	—	—	C	S	—	S	S	C	—	—	—
Vital records death	—	S	C	S	C	S	S	C	C	C	S
Clinic case reports	C	C	C	C	C	C	C	C	S	C	C
Electronic health records	—	U	—	—	—	—	—	—	U	—	C
Children with special health care needs data	C	—	—	—	—	C	—	—	—	—	—
Medicaid claims and enrollment	C	C	—	C	C	C	C	C	C	U	C
Hospital discharge data (some or all facilities)	S	C	—	C	C	C	C	C	C	S	—
LexisNexis data (for deaths)	S	—	—	—	—	—	—	—	—	—	—
Emergency medical services or emergency department encounter data	C	C	—	C	C	C	C	C	C	C	—
Ambulatory surgery (might be included in hospital discharge data in some states)	—	C	—	C	—	—	—	—	—	—	—
Medicare claims and enrollment	U	U	—	U	U	—	—	—	—	—	—
National Provider Index	—	S	—	—	S	—	—	—	—	—	—
State health benefit plan	—	—	—	C, U	—	—	—	—	—	—	—
Linked hospital, emergency department, and Medicaid	—	—	—	—	C	—	—	—	—	—	—
Immunization registry	—	—	—	—	U	S	—	—	—	—	—
All claims payer database	U	U	S	—	—	—	S	—	—	U	—
American Community Survey and U.S. Census Bureau data	—	S	—	—	S	—	—	S	—	—	—
Clinic laboratory data	—	—	—	—	—	—	—	S	—	—	—
Opioid prescription database	—	—	—	U	—	—	—	—	—	—	—
Education data	—	—	—	—	—	U	—	—	—	—	—
Private payer claims	—	—	—	—	—	U	—	U	—	—	—

**Abbreviations:** — = data sources that were determined to be lower priority or are yet to be explored by Sickle Cell Data Collection teams; C = core; S = supplemental; U = unavailable but desired.

Although different states initiated NBS for SCD at different times, all 11 SCDC states included NBS as a core data source to estimate SCD incidence, identify and confirm cases, and obtain sickle cell genotype or subtype information. Hospital and emergency department medical claims and encounter data that provide information on health care use, diagnoses, case location, and expected payer are planned for use as a core data source by nine states; this source was unavailable in two states (Colorado and Wisconsin).

State vital statistics (birth certificate data, death certificate data, or both) are accessed by 10 states and are being requested by an eleventh. Five states consider death data a core data source, and five consider it a supplemental data source. Three states used birth certificates as a supplemental data source. This determination of core versus supplemental depends on whether vital statistics are available and linked to other data sources within public health data warehouses.

As of 2021, all 11 participating states plan to use clinic data sets or case reports from hospitals or clinic electronic health records (EHRs) (as a core data set in 10 states and as a supplemental data set in one state). Clinic case reports are used to confirm case status and genotype and to identify who is being cared for by hematologists or other health care providers with experience caring for persons with SCD. Medicaid claims data sets serve as a core data source in nine states and are unavailable in one state.

## Discussion

### Prevalence Estimates and Population Characteristics

Although SCD is one of the most common inherited blood disorders in the United States, the number of persons living with SCD is unknown. Two national studies derived estimates using U.S. Census data, SCD prevalence among non-Hispanic Black and Hispanic persons (based on previously published estimates in one study and NBS data in the other study), and estimated life expectancy for children and adults with SCD. U.S. population prevalence estimates from these two studies were approximately 90,000 persons with SCD in 2005 in the first study (*32*) and approximately 100,000 persons with SCD in 2008 in the second study (*34*). Both studies provided state-specific estimates in addition to national estimates; these can be compared with the SCDC estimates for Georgia and California that are reported here, albeit with the caveat that the SCDC estimates are from a more recent period (2018). The 2018 annual SCDC prevalence estimate for Georgia of 9,141 cases is much higher than the estimates of 5,890 cases previously reported in the first study (*32*) and 4,981–5,797 cases previously reported in the second study (*34*). The 2018 prevalence estimates of 6,027 SCDC cases in California is similar to that reported by the first study (6,474 cases) (*32*) and higher than estimates reported by the second study (4,240–4,707 cases) (*34*).

Collecting, summarizing, and reporting multisource, population-based, longitudinal data for persons with SCD during 2004–2018 has provided important information about the demographics and characteristics of cases contributed by each source of data. Although California and Georgia have a similar percentage of cases ascertained from NBS and Medicaid data sources, they have markedly different age distributions of their SCD population as well as diversity in the number of clinic sites reporting. NBS programs contributed 16% of the overall cases of SCD to the surveillance system in California and 17% in Georgia over the 15-year period. During 2004–2018, clinic reporting accounted for 28% of the total cases identified in California and 56% in Georgia. The lower percentage in California is consistent with the greater number of clinic sites in California reporting a smaller number of cases and the challenges California has in engaging the entire network of SCD providers in surveillance activities. These percentages also highlight the substantial number of cases that would be missed if SCD surveillance relied solely on clinical disease registries to identify cases because many SCD patients lack access to centers providing comprehensive SCD care.

During 2004–2018, in California and Georgia, 64% of the cases were found in the Medicaid data, suggesting that approximately two thirds of persons with SCD were publicly insured during a certain part of their lives. Of cases identified in the Medicaid data, 31.6% were confirmed (2,058 of 6,499) in California, and 65.1% were confirmed (6,184 of 9,503) in Georgia. In addition, 87% of the cases in Georgia and 71% in California were found in the hospital or emergency department claims data. Medicaid or CHIP data and hospital or emergency department claims data can be used longitudinally to study trends over time in outpatient, inpatient, and pharmacy use for Medicaid enrollees and acute health care use for all patients, respectively.

SCD subtype or genotypes of confirmed cases were similar across states and data sources and consistent with previous research. Sickle cell anemia accounted for 63% of the cases in both states, followed by HbSC (26%–28%), HbS/β^+^-thalassemia (7%–8%), and other compound heterozygous forms of SCD (1%–3%). Males and females were equally represented in the confirmed cases; however, females were overrepresented in the probable cases, likely because females have more health care use during their child-bearing years and are more likely to be covered by Medicaid (*37*). Differences were observed in the age distribution of patients identified by each data source and across states.

### Findings for Health Care Providers and Policymakers

SCDC has been ongoing since 2015 in California and Georgia with both programs continually engaging their community (community-based organizations and patient advocacy groups), public health (state public health department and NBS), clinical, and federal partners through statewide, multidisciplinary stakeholder meetings to help guide the focus, content, and information dissemination for the project. Examples of collaboration using SCDC data through community engagement include geographic analyses indicating where persons with SCD live in relation to SCD specialty health care providers or facilities and analyses of trends in recommended health care treatments (e.g., hydroxyurea).

Geographic assessments in both Georgia and California apprised decision makers on the need for additional community resources and SCD specialty care in the respective states. For example, California’s SCDC data were provided to policymakers in advance of a vote on a bill to fund care centers, community health workers, and professional workforce development to better serve adults living with SCD covered by Medicaid in the state. The $15-million, 3-year program, Networking California for Sickle Cell Care, was funded in 2019 (*38*). In Georgia, SCDC data and geographical maps supported SCD community and patient advocates who provided testimony to the State Senate Committee on Sickle Cell Anemia highlighting gaps in access to care and lack of specialized care for persons with SCD outside of metropolitan Atlanta and Augusta. As a result, the state senate finalized recommendations for the care of persons with SCD, including the use of existing Federally Qualified Health Centers and establishment of additional mobile units to increase care. Other recommendations focused on improving provider training in SCD through Georgia medical schools and primary care residency programs. A pathway for extending Medicaid benefits for persons aged 18–25 years with SCD, regardless of Supplemental Security Income insurance or disability status, also was recommended (*39*). Additional information about the geographic distribution of SCD clinics in California and Georgia is available (Supplementary Figures 1 and 2, https://stacks.cdc.gov/view/cdc/120911).

Findings on hydroxyurea use were shared with health care providers. Although hydroxyurea use among Medicaid beneficiaries with SCD who live in California or Georgia increased, many beneficiaries with severe complications of SCD do not use hydroxyurea, highlighting the need to address concerns about safety, adherence, and access to care, among other barriers. Further information on use of hydroxyurea among persons with SCD in California and Georgia is available (Supplementary Material 2, https://stacks.cdc.gov/view/cdc/120911).

Surveillance data also have enabled the development of multiple educational materials for patients and families (e.g., videos, infographics, fact sheets, webinars, and social media content), equipping patients to be more active in decisions about their care. A provider-level educational webinar series to improve patient care also has been developed. Data also have prompted new research initiatives for the disease. Additional information on the utility of SCDC data in California and Georgia is available (Supplementary Material 3, https://stacks.cdc.gov/view/cdc/120911).

### SCD Surveillance Process Assessment

The data from Georgia and California provide important information on health care and outcomes of persons with SCD in each state, although drawing conclusions about national outcomes requires more information about patients across the country because health care systems and patient populations vary by state. The challenges that patients face in one state might not be the same in other states and, consequently, recommendations for interventions to improve health outcomes also might be different.

On the basis of the performance and strengths of the SCDC California and Georgia programs, CDC implemented 1-year SCDC capacity-building projects in seven additional states in 2019. In 2021, two more states were added to the SCDC network when funding became available, bringing the total to 11 states participating in SCDC.

CDC, in partnership with the established SCDC programs in California and Georgia, conducted a series of capacity-building sessions to help other states develop rigorous SCDC programs. Findings from the SCDC process assessment, which was first implemented in 2020 upon completion of the capacity-building sessions, suggest there is no consistent “cookbook” approach to building these systems. States had different systems for collecting administrative data and different capacities to gain access to data sources, such as hospital discharge and Medicaid data. In addition, the multidisciplinary teams convened by each state varied in leadership, from health care providers and university researchers to government agencies. Capacity and cost to implement and sustain SCD surveillance also differ widely among these settings. Nonetheless, methods for standardizing reporting on disease prevalence, geography, health care use, and deaths were developed across all participating states, and the process assessment indicated that expansion of SCDC to multiple states is feasible.

As additional states join the SCDC program, data sources for case identification are being expanded to include EHRs and all payer claims databases. Supplementary data sources of interest include school, immigration, and employment records and data obtained through surveys or biosampling of persons with SCD. Although not incorporated into SCDC, these data sources provide opportunities for expansion or one-time studies to answer specific research or policy questions.

## Limitations

The findings in this report are subject to at least three limitations. First, SCDC relies on administrative data from public payors (Medicaid) and hospital discharge data (inpatient and emergency department claims) to identify probable cases using SCD diagnosis codes. Therefore, SCDC case ascertainment methods might miss persons who do not access hospital-level care, were born before NBS for SCD, or are uninsured or privately insured. Although this limitation would lead to underestimates of prevalence when limited years of data are included, it becomes less limiting when surveillance includes more years of longitudinal data because most adults with SCD eventually access acute care. Second, SCDC cannot track persons with SCD if they have moved from their home state to another because of challenges in accessing data, including NBS records. Further, SCDC cannot capture health care use that might occur in hospitals outside of the state, particularly for persons living near the state borders. This lack of real-time tracking of patient addresses could lead to overestimating period prevalence, which is why annual reports attempt to adjust for patient migration by requiring persons to have evidence of in-state health care use during the year or before and after the year in question. Finally, SCDC cannot provide real-time information on persons with SCD because it is a passive surveillance system. Because timeliness of data is based on availability of administrative data before the core data set can be linked and cleaned, the data available for analyses and reporting are usually ≥2 years old. Timeliness of the data is particularly limiting for assessing the impact of recent acute events on SCD outcomes, such as a pandemic, a new therapy, or a recent practice change. Despite these limitations, the collaborative infrastructure that SCDC programs continue to develop, both within their states and across the SCDC network, provides opportunities to consider novel and innovative approaches to assessing data completeness and enhancing case ascertainment.

## Future Directions

With sufficient resources, SCDC could continue to track patient-level data from multiple sources about persons living with SCD. As additional funding becomes available, SCDC will expand to new states and allow comprehensive surveillance of those living with SCD because every state has a unique demographic makeup and distinct health care policies, medical and research centers, and access to care. For SCDC to be relevant and useful, states will need to focus on continually engaging their stakeholders to ensure data collection and analyses address the priority needs of the community, including addressing gaps in population-based research, suggesting clinical practice guidance, providing data needed for health care services assessment and planning, and prioritizing patient-provider education needs. An ongoing interest for SCDC is to build a more robust system that will provide a platform for linking surveillance data with disease registries within and across states. These data systems, although not mutually exclusive, will provide complementary information. The proposed robust system will allow for the simultaneous study of improvements in quality of care for persons who routinely seek and receive care from an SCD specialist (which include hematologists as well as other health care providers with experience caring for persons with SCD) and access to care for those who are not connected to coordinated SCD care or to an SCD specialist. This will create opportunities to develop policies and improve care-navigation programs, including health care transition programs and outreach to persons with SCD who will benefit from routine health management programs with their local primary care providers. SCDC will also work to address two data challenges: 1) identifying approaches to data aggregation and assessment to determine what data should and should not be aggregated across states and 2) building a more robust platform that will allow for tracking across states.

## Conclusion

This report describes SCDC activities and provides a framework for states or other entities planning or implementing surveillance programs for SCD. The case-finding methods used by SCDC provide the most comprehensive state SCD prevalence estimates available. SCDC incorporates data from multiple sources rather than collecting data from selected health care facilities or single data sources. This method is able to capture cases of SCD within a state, regardless of where patients live or where they receive care within the state. However, additional efforts are needed to improve the timeliness, completeness, and validity of case ascertainment, such as by adding Medicare claims, incorporating EHR data, and validating case definitions within adult populations. Continued support of the SCDC program will enable states to collect additional years of longitudinal data, which will allow for further refinement of prevalence estimation methods.

Although there are differences among funded SCDC programs, all participating SCDC state teams have access to robust data sources that will form the foundation of comprehensive population-based surveillance in their respective states. SCDC surveillance efforts have established disease prevalence in California and Georgia and have substantially advanced understanding of the health care needs of the population living with SCD in those states. SCDC teams in newly funded states are likewise committed to engaging with stakeholders and motivated by the utility of the collected and linked data for improving care and outcomes for persons with SCD and tracking those improvements over time.

## Footnotes

* 45 C.F.R. part 46.102(l)(2), 21 C.F.R. part 56; 42 U.S.C. §241(d); 5 U.S.C. §552a; 44 U.S.C. §3501 et seq.
